# Swarm Intelligence Internet of Vehicles Approaches for Opportunistic Data Collection and Traffic Engineering in Smart City Waste Management

**DOI:** 10.3390/s23052860

**Published:** 2023-03-06

**Authors:** Gerald K. Ijemaru, Li-Minn Ang, Kah Phooi Seng

**Affiliations:** 1 School of Science, Technology & Engineering, University of the Sunshine Coast, Moreton Bay Campus, 1 Moreton Parade, Petrie, QLD 4502, Australia; 2 School of AI and Advanced Computing, Xian Jiaotong Liverpool University, Suzhou 215123, China

**Keywords:** swarm intelligence, Internet of Vehicles, wireless sensor networks, smart city, waste management

## Abstract

Recent studies have shown the efficacy of mobile elements in optimizing the energy consumption of sensor nodes. Current data collection approaches for waste management applications focus on exploiting IoT-enabled technologies. However, these techniques are no longer sustainable in the context of smart city (SC) waste management applications due to the emergence of large-scale wireless sensor networks (LS-WSNs) in smart cities with sensor-based big data architectures. This paper proposes an energy-efficient swarm intelligence (SI) Internet of Vehicles (IoV)-based technique for opportunistic data collection and traffic engineering for SC waste management strategies. This is a novel IoV-based architecture exploiting the potential of vehicular networks for SC waste management strategies. The proposed technique involves deploying multiple data collector vehicles (DCVs) traversing the entire network for data gathering via a single-hop transmission. However, employing multiple DCVs comes with additional challenges including costs and network complexity. Thus, this paper proposes analytical-based methods to investigate critical tradeoffs in optimizing energy consumption for big data collection and transmission in an LS-WSN such as (1) finding the optimal number of data collector vehicles (DCVs) required in the network and (2) determining the optimal number of data collection points (DCPs) for the DCVs. These critical issues affect efficient SC waste management and have been overlooked by previous studies exploring waste management strategies. Simulation-based experiments using SI-based routing protocols validate the efficacy of the proposed method in terms of the evaluation metrics.

## 1. Introduction

One of the most critical services offered in the smart city (SC) framework is IoT-enabled waste management; data collection and traffic engineering are critical aspects of this framework in the context of waste management scenarios. Provision of efficient waste management is becoming challenging due to rural–urban drift and the fast growth in the urban population, which has given rise to large cities. To meet the requirements of the residents of such cities, there is a need to offer efficient waste management services. An emerging approach to the provision of efficient waste management in this context has been linked to methods of data collection and transmission for waste management operations. Current techniques exploring a single mobile element with IoT-enabled technologies for data collection and transmission for waste management scenarios are no longer sustainable due to the emergence of sensor-based big data systems and large-scale wireless sensor networks (LS-WSNs), which require network partitioning into multiple groups of clusters and regions prior to data collection. Thus, it has become expedient to devise new techniques for energy-efficient data collection and transmission to optimize energy consumption for big data collection for SC waste management strategies. 

Waste management strategies entail all activities and operations relating to waste, including the collection, handling, transportation, and proper disposal at appropriate locations. According to [1], waste management strategies involve a set of processes ranging from collection of waste to the recycling of the collected waste. Waste management is therefore a critical component of an SC framework. A comprehensive review of SC waste management techniques can be found in [2]. Several studies on waste management strategies have focused on exploiting IoT-based technologies. However, this is associated with the challenge of high energy consumption of sensor nodes, which limits the operational lifetime of the network. Thus, optimizing energy consumption and operational costs to meet the energy requirements of the network is a major focus of the current research approach. 

Realizing the harmful effects of improper refuse disposal on the environment, it has become necessary to devise efficient waste management strategies for SC scenarios. Furthermore, owing to the surge in urban population growth, waste management data are growing astronomically and are projected to account for 70 percent of the Earth’s population by 2050 [3], giving rise to large cities. Such cities are required to be ‘smart’ to meet the demands of citizens by offering advanced and efficient services including smart waste management. Thus, efficient waste management strategies are a critical aspect of an SC framework. However, new challenges associated with energy-efficient waste management have emerged due to the need for sensor-based big data collection architectures and the emergence of large-scale wireless sensor networks (LS-WSNs) in smart cities. Thus, the current traditional IoT-based methods for data collection and transmission for waste management are no longer sustainable in the context of SC waste management scenarios because the sensors are battery-operated, with several limitations on memory capacity, processing, and storage capabilities to hold large volumes of waste management data that characterize an SC ecosystem. Because data collection and transmission operations are energy-dependent, network operations will be hampered if data transmission is impacted by energy-deficient sensor nodes. Hence, this paper proposes a novel energy-efficient swarm intelligence IoV-based method for opportunistic data collection and traffic engineering for SC waste management. This method involves deploying multiple data collector vehicles (DCVs) traversing the entire network to gather sensed data via a single-hop transmission for SC waste management operations. None of the existing waste management strategies has considered this approach to optimize energy consumption for big data collection and transmission in an LS-WSN.

Previous studies have shown multiple ways by which sensor data can be transmitted from sensors to the base station (BS) or access point (AP). For a waste management scenario, the traditional method involves data transmission from sensors directly to the sink for processing via a multi-hop communication formed by the sensor nodes. Multi-hop communication enables nodes near the sink node to relay data from the nodes that are farther way from the sink node. While this approach may be feasible for data transmission, it creates some bottlenecks in the network. The major drawback is the energy-hole problem created as a result of frequent data-forwarding activities of the sensor nodes that are near the sink node [4,5,6]. Such nodes usually dissipate their energies faster than their counterparts that are stationed farther away from the sink node. Hence, multi-hop data-forwarding strategy can be ineffective due to the energy-hole problem occasioned by frequent data forwarding resulting in high transmission overheads [2,7]. Furthermore, the emergence of LS-WSNs with sensor-based big data systems makes this approach inefficient for SC waste management strategies. 

Inspired by the breakthroughs in recent studies that have shown the efficacy of mobile elements [8,9,10,11,12] over traditional static multi-hop routing, this paper proposes a novel energy-efficient swarm intelligence (SI) IoV-based model exploiting multiple DCVs for opportunistic data collection and traffic engineering for SC waste management. We propose analytically based methods to investigate some of the critical issues affecting efficient data collection and transmission for SC waste management strategies, which include: (1) determining the optimal number of data collector vehicles (DCVs) required in the network and (2) determining the optimal number of data collection points (DCPs) for the DCVs. Previous studies exploring waste management strategies have not considered this approach to optimize energy consumption for big data collection. The focus of this paper is the deployment of vehicular networks for opportunistic data collection and traffic engineering for waste management operations in an SC. The proposed approach aims to address the two major challenges of efficient data gathering and transmission for DCVs in large-scale wireless sensor networks (LS-WSNs) in SC scenarios. We also consider the challenge of data collection from spatially separated regions (unconnected regions), which requires network partitioning into multiple sizes and groups prior to data collection. This issue poses a great challenge that makes it infeasible to deploy a single DCV for data-gathering activities, since a single DCV does not have the energy capacity to traverse the entire network for the purpose of data gathering and delivery to the BS or AP. Based on this scalability issue with a single DCV in an LS-WSN scenario, this work considers exploiting multiple DCVs to minimize latency times since there may be many data collection points (DCPs) to be visited. Thus, the proposed approach leverages the advantages inherent in the use of multiple mobile collectors to address some of the limitations of the single data collector scheme. However, employing multiple DCVs comes with additional challenges including costs and network complexity. Hence, there is a need to create a balance to guarantee network stability by determining the optimal number of DCVs required for continuous network operation. 

The objective of this paper is to fill the research gap in this area by proposing a novel SI IoV-based model utilizing vehicular networks to address the critical tradeoffs affecting efficient big data collection and transmission in an LS-WSN for an SC waste management scenario, which include: (1) finding the optimal number of data collector vehicles (DCVs) needed in the network, (2) finding the optimal number of data collection points (DCPs) for DCVs, and (3) partitioning the network into regions of multiple sizes and assigning a DCV to each region. We note that this approach represents a significantly delay-tolerant network (DTN) that employs different data delivery mechanisms including exploitation of vehicles for opportunistic data collection and transmission. Below, we further highlight the major contributions of this, work which are explained as follows:

(1) Due to issues of network complexity and cost implications as a result of the deployment of multiple data collector vehicles (DCVs), it is expedient to determine an appropriate number of DCVs. Thus, we employed an analytical approach to determine the optimal number of DCVs required in the network within a given time period, including the optimal trajectory for each DCV;
(2) We considered the optimal number of data collection points (DCPs) for the DCVs, including their locations and data collection times at a given location;
(3) For LS-WSN scenarios exploiting multiple DCVs, we first consider the need to partition the network into multiple finite regions using the improved *k-means* algorithm and assign each region to a DCV using the principle of distance matrix between OPs and DCVs;
(4) Simulation experiments using swarm intelligence algorithms are further provided to validate the analytically based method.

The rest of this paper is structured as follows. Section 2 presents an IoV-based architecture for SC waste management strategies, and related works are presented in Section 3. Section 4 presents the system model and problem formulation. The proposed work is presented in Section 5. Section 6 presents simulation results and analysis, while Section 7 concludes the work. 

## 2. IoV-Based Architecture for SC Waste Management Strategies 

**Figure 1:**
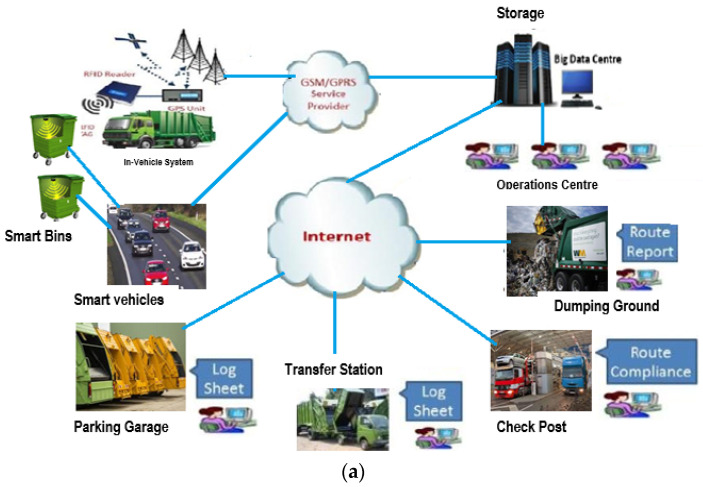

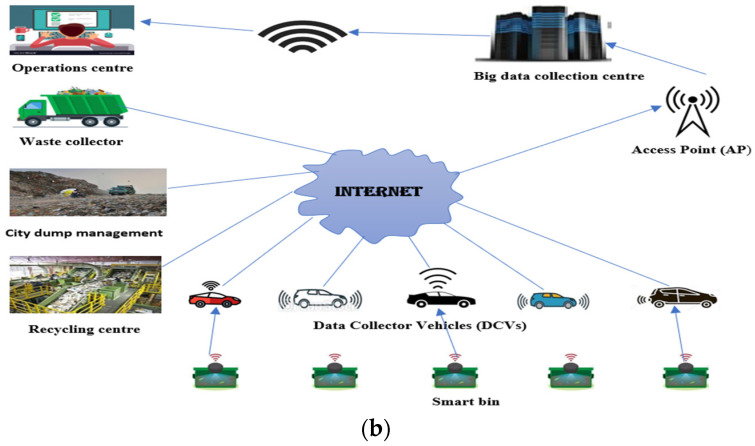
An IoV-based network model for smart city waste management. (**a**) An overview of Internet of Vehicles smart city waste management. (**b**) A model showing data collection for SC waste management.

The connectivity between vehicles and the Internet of Things (IoT) has led to a new era of technological advancements in terms of vehicular networks known as the Internet of Vehicles (IoV), which depicts a global convergence of the traditional IoT and the mobile internet [13], whereby vehicles serve as smart moving nodes in the network. The authors of [2,14,15], conceive of the IoV as a technological platform that enables a network of interactions between vehicles and other devices or objects within their environment, which may include other vehicles, sensors actuators, humans, and road infrastructure. IoV-based technology provides a huge network of interactions amongst the various communication models exploiting IoT-based technologies to enable big data information on smart waste bins scattered within an SC ecosystem and the collection and transmission of such data using vehicular networks. Some application specifics of IoV for SC solutions can be found in the areas of big data analytics, infotainment, intelligent transportation systems, cloud computing, crash response, environmental management, and traffic management [13,14,15,16,17,18]. Recent studies on IoV reveal that researchers have focused on the above-mentioned application specifics but have yet to extend this application to SC waste management. Other studies show that research efforts are currently focused on exploiting IoT-enabled techniques for SC waste management strategies. Hence, the potential of the IoV has not yet been explored for SC waste management solutions. One of the challenges of using IoT-enabled technologies for data collection for waste management is the high energy dissipation of the sensor nodes, which is occasioned by high transmission overheads. To optimize energy consumption of sensor nodes, many researchers have proposed the use of mobile collectors for data collection [6,7,8,9,10,19]. Thus, this study aims to harness the potentials inherent in IoV-based methods for an intelligent waste management strategy in SCs. In this study, we further investigated some of the critical issues and tradeoffs affecting efficient data collection and transmission for SC waste management solutions using analytically based approaches. Figure 1 shows the architecture of IoV-based waste management in an SC. While Figure 1a gives an overview of IoV-based SC waste management, Figure 1b presents a model of data collection for an SC waste management technique. 

We note that several research efforts have utilized multiple mobile elements for data collection [20,21,22] to tackle the challenges of scalability associated with a single collector scheme. However, none has considered an IoV-based approach to address the need to optimize energy consumption for big data collection in LS-WSNs by examining critical issues and tradeoffs affecting efficient data collection for SC waste management solutions. Some advantages of exploiting vehicles as mobile collectors are that (1) vehicles are able to traverse and roam across different unconnected geographic regions of the SC for data collection and transmission purposes; (2) vehicles can be used to optimize energy consumption of sensor nodes by enabling short-range data collection and transmission, which saves substantial energy use by sensor nodes and minimizes their transmission overheads; (3) unlike the energy-constrained IoT-based sensors, vehicles are energy-renewable; (4) vehicles can handle the issues of flexibility and scalability associated with a typical single collector scheme; and (5) the opportunistic approach enables the same vehicle to be simultaneously exploited for multiple applications to minimize energy consumption. 

**Figure 2:**
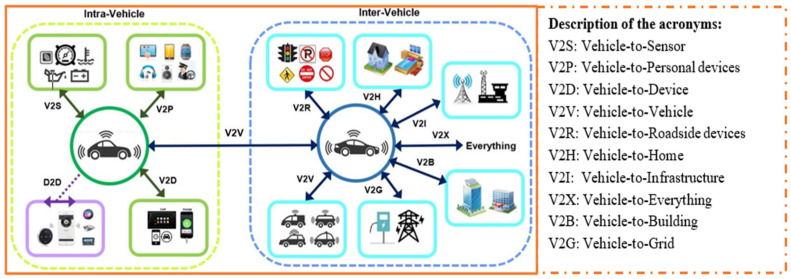
IoV-based network model showing V2X technologies.

**Figure 3:**
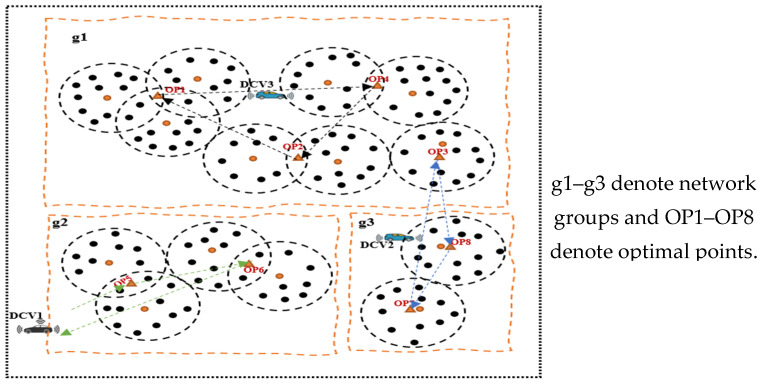
A working model of the proposed approach in an LS-WSN.

Despite the notable efficacies of multiple mobile collectors, especially in relation to the issues of scalability and latency, there are several challenges associated with the scheme that are related to costs and complexity of the network, thereby necessitating a tradeoff between network cost and delays. We consider the need for a timely data collection and transmission before the data loses its significance and becomes useless and therefore propose a novel energy-efficient swarm intelligence IoV-based approach for opportunistic data collection and traffic engineering for SC waste management. Figure 2 shows an IoV-based network communication model, while Figure 3 shows a working model of the proposed approach for data collection in an LS-WSN environment. The first challenge is partitioning the entire network into multiple clusters and groups prior to data collection. To this end, we adopted the network partitioning method presented in [11]. The other challenge is how to find the optimal data collection points (DCPs) for the DCVs and subsequently assign some DCPs to each DCV in a manner that tends to reduce the round-trip time for the *n* number of DCVs and consequently minimize their total movement energy consumption. Otherwise, some DCVs will have more DCPs to visit than others, resulting in high movement energy consumption as a result of longer tour duration. This work is focused on a network with predetermined locations of smart bins or cluster heads (CHs) and optimal DCPs such that the DCVs do not necessarily need to traverse all the CHs but can visit certain optimal locations for data gathering. 

## 3. Related Works

The challenges of efficient waste management, especially in urban cities, are becoming a critical issue that needs to be addressed headlong by devising appropriate waste management techniques to improve the environmental conditions and wellbeing of residents. In this section, we present a review of related works and draw comparisons with existing studies from two basic perspectives: (1) IoT-based waste management strategies in an SC ecosystem and (2) swarm-intelligence-based techniques. This section critically examines the proposed approach in comparison to other existing works and related studies. 

### 3.1. IoT-Based Waste Management Strategies in an SC Ecosystem 

This section discusses the application of smart technologies to solid waste management in an SC. Some methods involve the use of sensors, such as IR sensors, ultrasonic sensors, RFID tags, Wi-Fi, and GPRS for real-time data generation and gathering from smart bins that are spatially distributed at different locations. Table 1 presents a summary of previous works on IoT-based techniques for waste management in the smart city domain. A comprehensive survey of IoT-based approaches for waste management in an SC is presented in [2]. Findings from the literature reveal that researchers have focused attention on the use of IoT-based methods for data collection for SC waste management strategies, although none has considered the IoV-based approach for data collection for waste management in an SC. In contrast, our proposed method exploits vehicular networks (IoV) for data collection and transmission for efficient waste management in an SC. In terms of SC waste management, none of the existing approaches from the literature have considered the need to optimize energy consumption for big data collection in an LS-WSN. To this end, we propose the use of multiple data collector vehicles (DCVs) to optimize energy consumption for big data collection in an LS-WSN. However, we note that the use of multiple DCVs also comes with some challenges and therefore propose a swarm intelligence IoV-based method to address some critical issues associated with efficient data collection.

### 3.2. Swarm Intelligence (SI)-Based Approaches

This section discusses the use of SI, which represents a novel field of artificial intelligence (AI) that deals with the study of biological species characterized by behaviors that are self-organized, as seen in social living beings such as ants, fishes, birds, and termites. This field of AI has been increasing in popularity and is known for its potential to resolve various optimization problems. SI is currently widely utilized in different application domains. Compared to traditional methods, SI-based approaches present several advantages in terms of flexibility, scalability, robustness, energy efficiency, and data delivery performance [11,48,49]. However, a few drawbacks, as noted in [48,49], include the lack of theoretical analysis, as SI studies are experimentally based, and the fact that SI algorithms are generally time-consuming and can suffer from stagnation, with premature convergence to a local optimum. Despite the apparent drawbacks, the popularity of SI is growing at an alarming rate, which may be due to the increasing demand for smart optimization techniques in several businesses and engineering scenarios. SI-based methods can be employed to handle optimization, scheduling, planning, design, and management problems [48]. SI-solutions are used for formal, computationally complex algorithms. The authors of [49] discussed two representative SI-based algorithms: particle swarm optimization (PSO) and ant colony optimization (ACO). The authors of [49,50,51] presented a survey of representative SI algorithms to handle optimization problems. A comprehensive survey of nature-inspired and SI-based algorithms for wireless sensor networks is provided in [52,53]. The authors of [54] presented a review of SI-based algorithms for IoT-based solutions. Their study aimed to introduce a new level of intelligence to IoT-based systems, providing an understanding of some technical aspect of SI algorithms and their potential applications in IoT. Since SI is characterized by major features such as robustness, scalability, flexibility, and interoperability, it is expected that these will be critical in meeting the architectural requirements of SI IoT-based systems. Table 2 shows a comparison of the proposed approach with other SI-based approaches in the context of SC waste management applications. Findings from the literature reveal that none of the existing techniques has considered the use of an SI IoV-based approach for SC waste management applications. 

## 4. System Model and Problem Formulation 

### 4.1. Network Model and Assumptions

**Figure 4:**
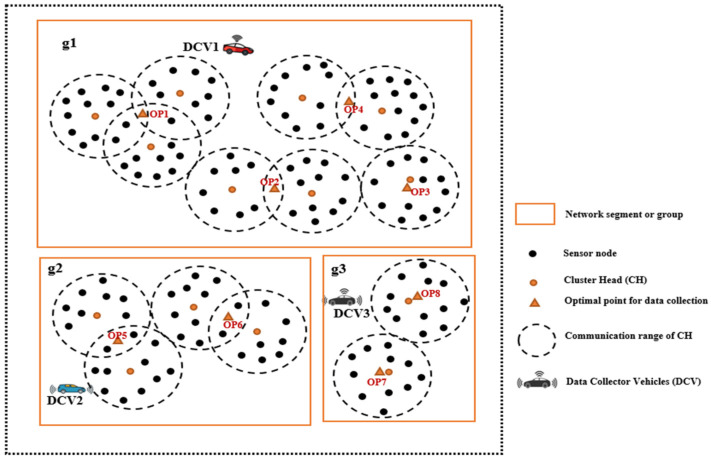
Network model and components for LS-WSN.

This section presents the network model of the proposed approach. Figure 4 shows the system model consisting of a large-scale wireless sensor network (LS-WSN) with a set of *N* stationary sensor nodes embedded in smart bins represented by a set ($N=\left\{N_{1},\;N_{2},\;N_{3},\;\ldots \;N_{n}\right\}$) and mobile data collector vehicles ($V=\left\{D_{1},\;D_{2},\;D_{3},\;\ldots D_{m}\right\}$). The network model consists of segmented group of clusters that are spatially distributed across different unconnected regions. There are three network groups (*g*$\left(g_{1},\;g_{2},\;g_{3}\right)$), and each group is unconnected to other groups. The network consists of a number of sensors, a set of cluster heads (CHs), and a set of optimal points (OPs) or data collection points (DCPs). CHs are leader nodes that collect data packets from other nodes and transmit them to the DCVs when they arrive at the DCPs. Data transmission to the DCVs occurs via a single-hop mechanism. The sensors in different groups or segments are clustered using the node partitioning algorithm presented in [11]. Each sensor is connected to the CH within its range of communication. Three major actors can be identified in the network, namely the static sensors (smart bins), the data collector vehicles (DCVs), and the base station (BS). The model makes the following assumptions:

(1) All the sensors and CHs are static after deployment;
(2) *N* number of sensors embedded in the smart bins are scattered across the smart city comprising different groups (*g*) and clusters with a set of optimal points. The groups are spatially separated and are not reachable by other groups. However, nodes within the same group are reachable by other nodes in their group and can communicate among themselves;
(3) Each DCV is a super-energy mobile collector that is not easily energy-deficient;
(4) All CHs are covered by the optimal data collection points (DCPs);
(5) The DCVs have knowledge of the locations of the CHs and OPs as they traverse the network, starting from the BS, which has the network information and makes it available to the DCVs;
(6) Each CH has adequate memory capacity to hold all the data that it transmits to the DCV upon arrival at an OP or DCP;
(7) The DCVs move at a fixed speed (*v*);
(8) The sensor nodes are randomly deployed a priori with known locations.

Table 3 presents a detailed list of symbols and their descriptions. 

The DCV starts its network tour from the BS and traverses along the designated OPs or DCPs to collect all the sensed data and finally returns to the BS for data offloading. Upon arrival at each region, the DCV communicates with the CH to collect all previously aggregated data. CH selection is dynamic, as every region reselects the CHs to minimize the hotspot problem and the data request flooding problem typical of multi-hop clustering. Data offloaded at the BS can be accessed by waste managers/authorities for decision-making purposes, including real-time routing and scheduling opertaions. For instance, waste collectors can be promptly scheduled and deployed for waste collection operations. 

Let Ć denote a set of *h* cluster heads (CHs) in a 2D Euclidean plane, i.e., $Ć=c_{1},\;c_{2}\ldots ,\;c_{h}$ with $\left(x_{i},y_{i}\right)$ coordinates of CH ${Ć}_{i}$ and 1≤i≤h. The neighborhood area of a CH (${Ć}_{i}$) is denoted by a circular disc ($P_{k}$), the center of which is *k* and the radius of which is $r_{k}$, where $r_{k}$ is a non-negative value. That is, $P_{k}$ is the set of cluster heads (including CH Ć) with coordinates (*x, y*) such that:

(1) $${\left(x-x_{i}\right)}^{2}+{\left(y-y_{i}\right)}^{2}\leq r_{k}{}^{2}$$

That is, $\{P_{k}\left|{\left(x-x_{i}\right)}^{2}+{\left(y-y_{i}\right)}^{2}\leq r_{k}{}^{2}\right|$, and Ć is a CH with coordinates (*x, y*)}. We note that any two discs, such as $P_{k_{i}}$ and $P_{k_{j}}$, may or may not overlap with each other. The DCVs visit the neighborhood regions of the CHs including the overlapped CH regions and optimal location points. It is also assumed that a DCV can receve data from a sensor lying within the vicinity and transmission range of the sensor (e.g., a 50 m range). Ignorance of this location point leads to a longer travel path, which results in large delays and an increase in energy consumption. Hence, it is expedient to determine the optimal DCPs for the DCVs to optimize energy consumption for big data collection in LS-WSNs. 

Let Ŕ denote a set of r regions such that $Ŕ=\left\{{Ŕ}_{1},\;{Ŕ}_{2},\ldots {Ŕ}_{r}\right\}$ and $\Omega_{OP}$ denote a set of *s* optimal points, i.e., $\Omega_{OP}=\left\{OP_{1},\;OP_{2},\;\ldots ,\;OP_{3}\right\}$. Let $D\left(OP_{j},\;OP_{j+1}\right)$ represent the distance between optimal points ($OP_{j},\;OP_{j+1}\in \Omega_{OP}$), and let $T\left(OP_{j}\right)$ be the time spent by a DCV stopping at $OP_{j}$. It is observed that the total time spent by a DCV on its tour is the sum of the traveling time and the stopping time at OPs in $\Omega_{OP}$. That is,

(2) $$T_{m}=\overset{s}{\underset{j=1}{\sum }}T\left(OP_{j}\right)+\overset{s}{\underset{j=1}{\sum }}\left(OP_{j},\;OP_{j+1}\right)$$

where $T_{m}$ denotes the total movement time of the DCV. We note that most of exisitng studies considered the traveling time of mobile collectors but overlooked the need to identify the optimal data collection points (DCPs) that tend to minimize the traveling time, data collection time, and path length. The optimal DCVs are identified by incorportaing both the total path length and the stopping time at optimal DCPs. 

Let *V* denote the optimal number of data collector vehicles. To access the OPs in $\Omega_{OP}$ by the *V* data collector vehicles, the set $\Omega_{OP}$ is divided into *V* disjoint subsets. Thus, ${\left\{OP_{i}\right\}}_{i=1}^{V}$ represents the partition of the entire set of Ops, where $OP_{i}$ has *n* number of OPs (i.e., $nOP_{i})$ such that $OP_{i}=\left\{OP_{i1},\;OP_{i2},\;\ldots ,\;OP_{inOP_{i}}\right\}$. 

### 4.2. Energy Model 

The energy model employed in the proposed work is the same as that presented in [59]. Mathematically, the energy dissipation of the network model can be represented using Equations (3) and (4). Equation (4) represents the energy consumption required to receive a *k*-bit data package.

(3) $$E_{tx}\left(k,\;d\right)=\{\begin{array}{c} k\left(E_{elec}+\varepsilon_{fs}d^{2}\right),\;if\;d<d_{0}\\ k\left(E_{elec}+\varepsilon_{mp}d^{4}\right),\;if\;d\geq d_{0} \end{array}$$

(4) $$E_{rx}\left(k\right)=k*\;E_{elec}$$

where *k* denotes the size of data packets in bits; *d* denotes the communication range between the transmitter and receiver; $E_{tx}$ and $E_{rx}$ denote the total energy spent on data transmission and reception, respectively; $E_{elec}$ is the radio electronics energy used by a sensor to transmit and receive a message; $\varepsilon_{mp}$ denotes the transmission amplifier energy in free space; $\varepsilon_{fs}$ denotes the factor of a multipath fading channel model; and $r_{0}$ denotes the threshold value and can be computed using Equation (5):

(5) $$r_{0}=\sqrt{\frac{\varepsilon_{fs}}{\varepsilon_{mp}}}$$

### 4.3. Problem Formulation

**Figure 5:**
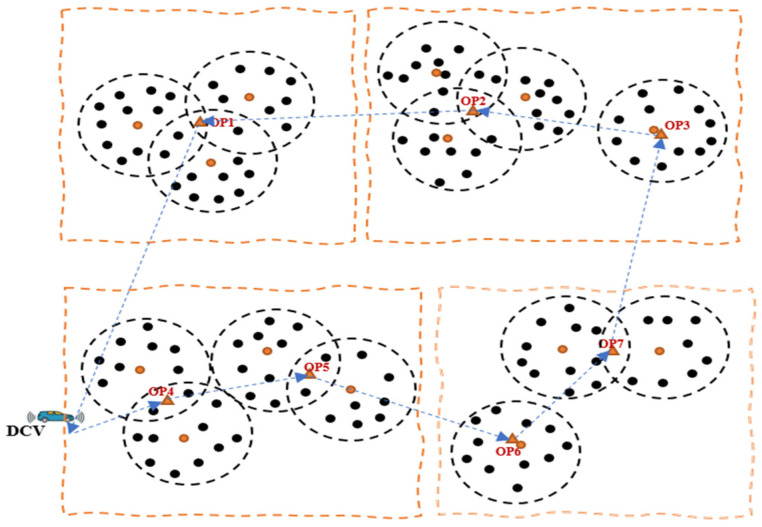
Optimal point selection and path computation with a single DCV.

**Figure 6:**
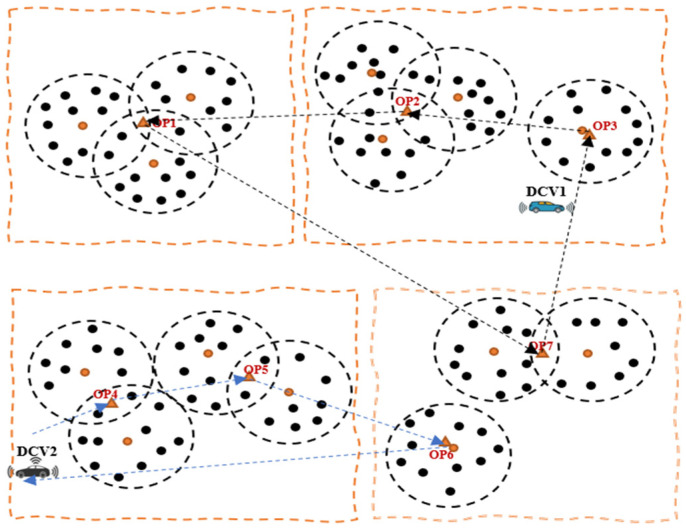
Optimal point selection and path computation with multiple DCVs.

For waste management in large-scale WSN scenarios, there are usually multiple data collection points (DCPs) scattered in different geographic areas. In this case, using a single data collector vehicle (DCV) for data collection from these locations is challenging and inefficient, since it will take a long time for the single DCV to traverse all DCPs, resulting in the incursion of delays and an energy-hole problem, creating operational bottlenecks in the network. In the context of waste management, this can create operational bottlenecks that can result in environmental hazards including air pollution, as most of the bins will have been overfilled before the deployment of waste collectors. Therefore, multiple DCVs will be required in such a situation to handle the scalability issues associated with a single DCV. However, employing multiple DCVs comes with additional challenges including costs and complexity of the network, thereby necessitating the need to create a balance between costs and delays by determining the optimal number of DCVs to stabilize network operations. One of the objectives of this study is to achieve this target to guarantee network stability and timeliness so that the data will not be useless. To this end, the DCVs are expected to follow the trajectory through optimal DCPs to minimize the data collection time and optimize energy consumption. We present the problem description in Figure 5 and Figure 6, which show an optimal point selection and path planning and computation with a single DCV and with multiple DCVs, respectively. 

## 5. Proposed Work

**Figure 7:**
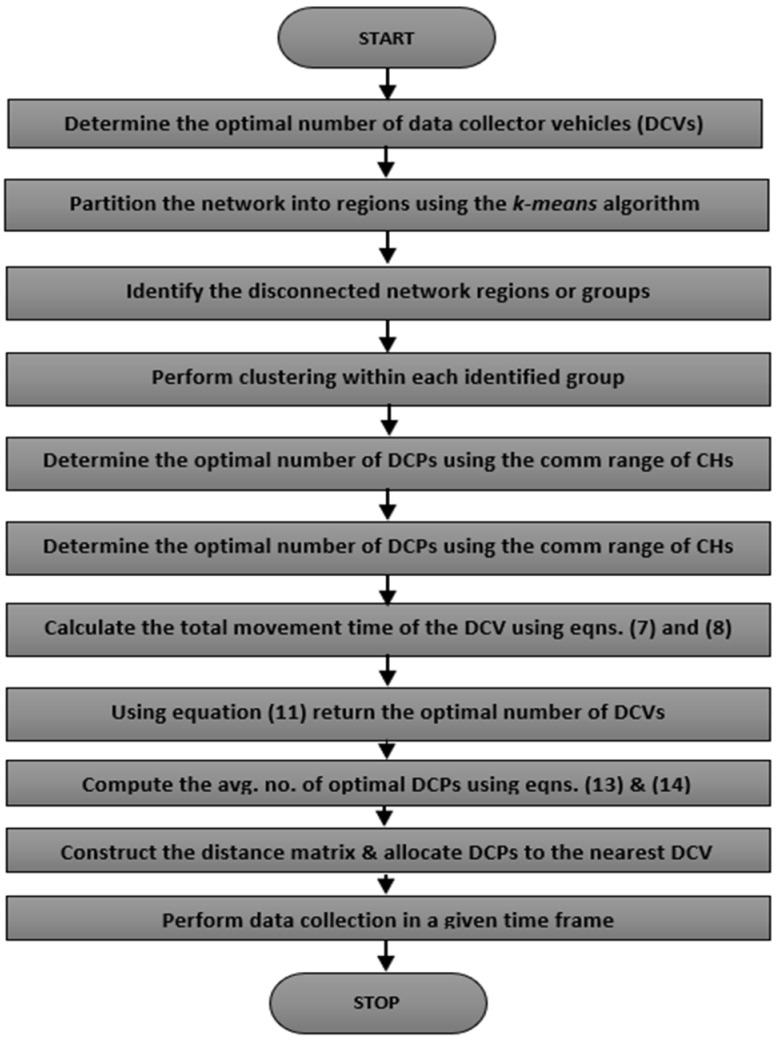
Flow chart of the proposed approach.

The proposed approach was used to investigate some of the critical tradeoffs affecting efficient big data collection and traffic engineering in LS-WSNs for SC waste management applications. The proposed scheme is an energy- and delay-efficient technique to identify the optimal number of DCVs and the optimal number of DCPs of DCVs. The purpose of this work is to leverage the advantages inherent in exploiting multiple opportunistic vehicles for data collection from multiple CHs simultaneously using a single-hop routing upon arrival at common overlapping regions of such clusters. Figure 7 shows the flow chart of the proposed model. 

### 5.1. Adaptive Network Partitioning Strategy

**Figure 8:**
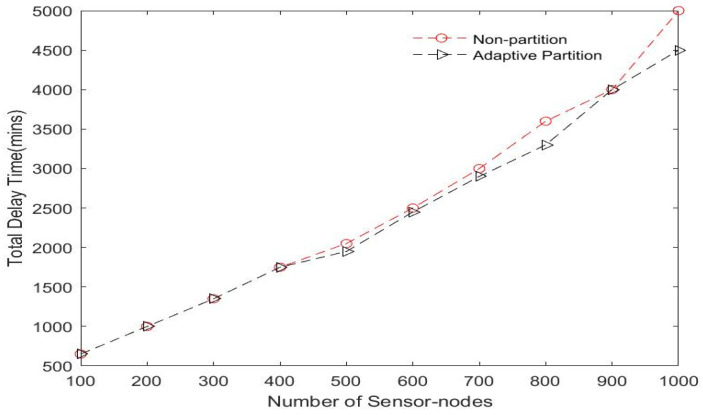
Comparing adaptive and non-adaptive partition schemes in terms of the number of sensor nodes.

**Figure 9:**
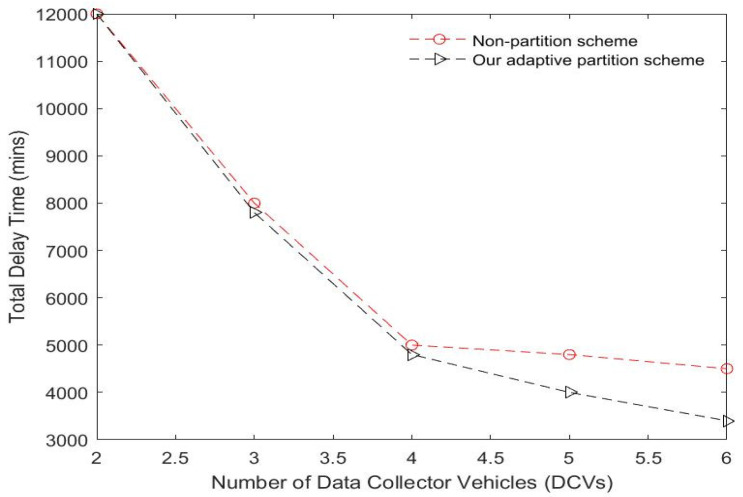
Comparing adaptive and non-adaptive partition schemes in terms of number of data collector vehicles (DCVs).

To effectively cover the widespread geographical regions of the network and optimize energy consumption for big data collection in an LS-WSN, we propose employing multiple DCVs. This provides advantages in terms of scalability, capability, flexibility, interoperability, and robustness. To solve the complex issue of scheduling multiple DCVs in LS-WSN scenarios, we propose an adaptive network partitioning strategy that partitions the entire network into multiple clusters and groups and assigns each group to a DCV. This partitioning scheme prevents DCVs from traveling long distances, thereby confining the scope of movements within the assigned regions. The use of a distributed clustering approach presents several advantages in real-world scenarios, as seen in [60], including energy consumption optimization, improving the route connection (i.e., minimizing delays), and increasing the scalability while reducing traffic. A brief description of the adaptive partitioning scheme is presented below. The network is adaptively partitioned into Ŕ regions, and the BS communicates this result to the DCVs. We adopted the improved *k-means* algorithm presented in [61] to perform network partitioning, which allows the DCVs to adaptively select a subset of sensor nodes with shorter distances from the centroid of each region. The objective is to minimize the traveling distance of the DCVs and optimize energy consumption. This is computed by taking the mean of (*x, y*) coordinates of all the nodes in the region. Nodes are assigned to the closest centroid of their region. Our adaptive network partitioning scheme adopted from [62] is compared to the traditional non-partitioning scheme for both small- and large-scale WSNs using some evaluation metrics such as energy consumption and delays. The results of this evaluation presented in Figure 8 and Figure 9 show that our adaptive network partitioning scheme is superior to the traditional method for LS-WSNs. However, the non-partitioning scheme appears to be effective for small-scale WSNs. 

### 5.2. Computation of the Optimal Number of DCVs 

This section presents an analytical approach to determine the optimal number of data collector vehicles (DCVs) needed to guarantee a continuous network operation. As stated earlier, in LS-WSNs scenarios, the DCVs may experience a very long traveling path for data gathering because many DCPs need to be traversed. Data collection in this case will incur large delays with a single DCV, especially for time-sensitive applications including waste management. For this reason, we propose the use of multiple DCVs since a single DCV is infeasible in an LS-WSN scenario. We aim to determine the optimal number of DCVs while ensuring network stability and timeliness. For the purpose of analysis, we plan to achieve this objective under a given time constraint ($T_{c}$). However, the approach can also be applied for delay-tolerant applications since we are using opportunistic mobile DCVs. If $T_{d}$ denotes the total time spent by a DCV in one trip of data gathering, then $T_{d}$ equals the total movement time on a fixed path plus the stopping time at optimal points for data collection. Thus,

(6) $$T_{d}=\overset{s}{\underset{k=1}{\sum }}T_{k}+\overset{s-1}{\underset{k=1}{\sum }}d\left(OP_{k}-OP_{k+1}\right)/v\;$$

where $T_{k}$ denotes the stopping time at the *k*th optimal point, and $d\left(OP_{k}-OP_{k+1}\right)$ is the Euclidean distance between two optimal points. Thus, the stopping time at an OP ($T_{so}$) is calculated as:

(7) $$T_{so}=\overset{\left|L\left(OP_{j}\right)\right|}{\underset{k=1}{\sum }}\overset{\left|N\left(l_{k}\right)\right|}{\underset{i=1}{\sum }}D_{k}/v\;$$

where $D_{k}$ denotes the data size of the CH ($l_{k}$), while $L\left(OP_{j}\right)$ and $N\left(l_{k}\right)$ denote the set of CHs and set of sensors within the $OP_{j}$ and CH $l_{k}$, respectively. The DCV moves at a speed of v (m/s). The stopping time of the DCV is negligible compared to the time spent for data collection. The time spent for data collection depends on the data size of the CH transmitting the data at every DCP. $r^{\prime }$ represents data collection rate of the DCV and *L_DREQ_* denotes the data size (i.e., length of the data request (DREQ) in bits). By inputting Equation (7) into (6), the DCV’s traveled time (i.e., the total time spent visiting all optimal points in a region) can be modified as follows:

(8) $$T_{d}=\overset{s}{\underset{k=1}{\sum }}\left(\overset{\left|L(OP_{k})\right|}{\underset{j=1}{\sum }}\overset{\left|s\left(l_{j}\right)\right|}{\underset{i=1}{\sum }}L_{DREQ}/r^{\prime }\right)+\overset{s-1}{\underset{k=1}{\sum }}d\left(OP_{k}-OP_{k+1}\right)/v\;$$

Let $T_{c}$ denote the deadline time constraint for data collection, and let *V* represent the optimal number of DCVs in deadline time constraint. Thus, the optimal time ($T_{opt}$) using *V* number of DCVs is given by

(9) $$T_{opt}=\frac{1}{V}\left[\overset{s}{\underset{k=1}{\sum }}\left(\overset{\left|L(OP_{k})\right|}{\underset{j=1}{\sum }}\overset{\left|s\left(l_{j}\right)\right|}{\underset{i=1}{\sum }}\frac{L_{DREQ}}{r^{\prime }}\right)+\overset{s-1}{\underset{k=1}{\sum }}\frac{d\left(OP_{k}-OP_{k+1}\right)}{v}\;\right]\;$$

For a deadline time constraint, the value is

(10) $$T_{opt}\leq T_{c}$$

Combining Equations (8)–(10), we obtain:

(11) $$V\geq \left[\frac{\sum_{k=1}^{s}\left(\sum_{j=1}^{\left|L(OP_{k})\right|}\sum_{i=1}^{\left|s\left(l_{j}\right)\right|}\frac{L_{DREQ}}{r^{\prime }}\right)+\sum_{k=1}^{s-1}\frac{d\left(OP_{k}-OP_{k+1}\right)}{v}}{d_{TC}}\right]\;$$

Hence, Equation (11) can be used to determine the optimal number of DCVs deployed in the network for data collection for SC waste management operations. We note that the initial position of each MCV depends on the limit of the deadline time of data collection and the path length. Based on the above parameters, the path length traveled by each $DCV_{i}$ ($L_{path\left(\vartheta i\right)}$) is calculated as:

(12) $$L_{path\left(\vartheta i\right)}=T_{c}\times v\;$$

**Figure 10:**
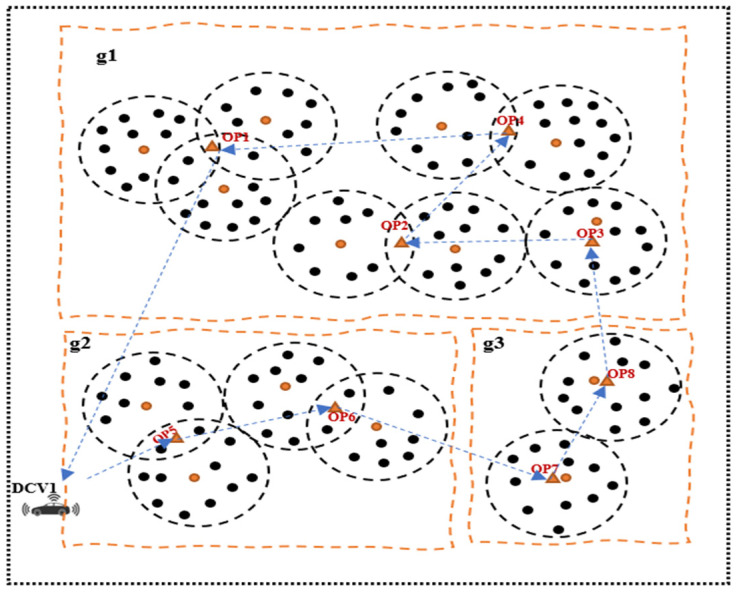
A working example of the proposed model using a single DCV.

**Figure 11:**
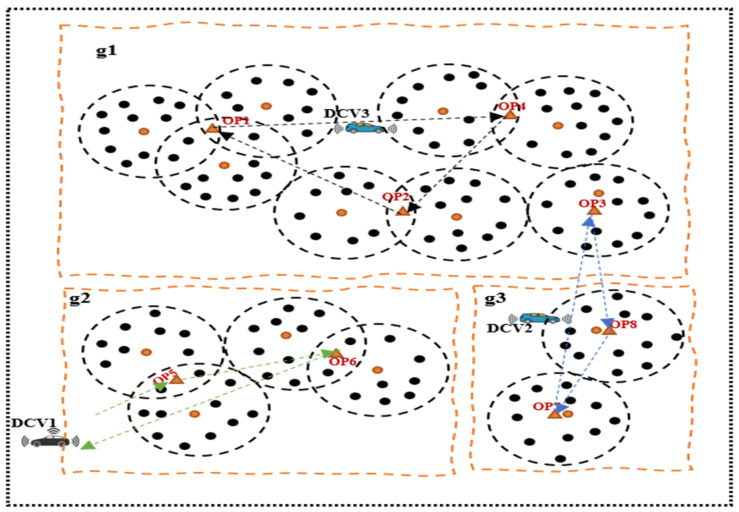
A working example of the proposed model using multiple DCVs.

To provide further clarification, if we consider a network containing 8 OPs and 13 CHs (i.e., leader nodes), as shown in Figure 10 and Figure 11 for a single DCV and multiple DCVs, respectively, and assume that each sensor relays 5 packets with a data size of 512 bytes, then CHs 1-13 have 50, 55, 65, 65, 60, 55, 65, 65, 100, 60, 50, 65, and 60 data packets to relay, respectively. If we set the data collection rate of each DCV from the CH to 40kbps, then the following scenarios will play out: (1) A single DCV moving at a speed of 2 m/s is used for data collection. The DCV begins its tour from the initial location and visits the closest OP to the CH for data collection from, i.e., OP5. The leader node at OP5 has 96 data packets for transmission, and the stopping time of the DCV to collect all the packets at OP5 is 10.92s according to Equation (7). Similarly, the stopping time of the DCV as it traverses along the other closest points (i.e., OP6, OP7, OP8, OP3, OP2, OP4, and OP1) can be computed using Equation (7) as 19.57 s, 15.87 s, 12.96 s, 9.50 s, 13.28 s, 11.54 s, and 10.78 s, respectively. Thus, the total stopping time is 104.42 s. The trajectory time as predetermined for 500 sensors is 980.96 s, in accordance with Figure 10. Hence, the total time for data collection and wireless charging equals the trajectory time plus the stopping time, i.e., (980.96 + 104.42) = 1085.38 s. Assuming the deadline time for data collection is 400 s, then we can compute the optimal number of MCVs based on Equation (11) as $V=\left(\frac{1085.38}{400}\right)=2.7≅3$.

In real-world applications, waste management data need to be retrieved and offloaded as quickly as possible to minimize environmental hazards arising from delays in scheduling and routing of waste collectors by decision makers. The performance of the proposed approach was tested using Equations (7)–(11), which demonstrate how to compute the optimal number of DCVs. Here, we computed *n* = 3 at 400 s.

**Figure 12:**
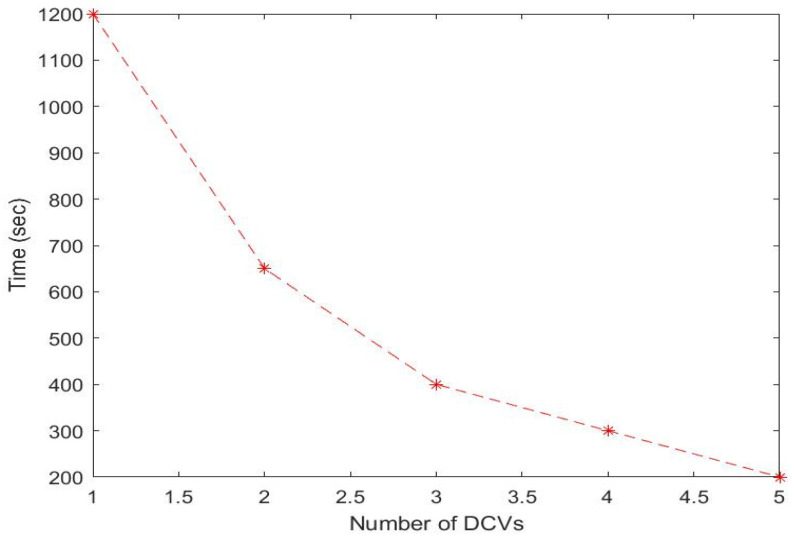
Maximum time usage with different numbers of DCVs from one to five.

**Figure 13:**
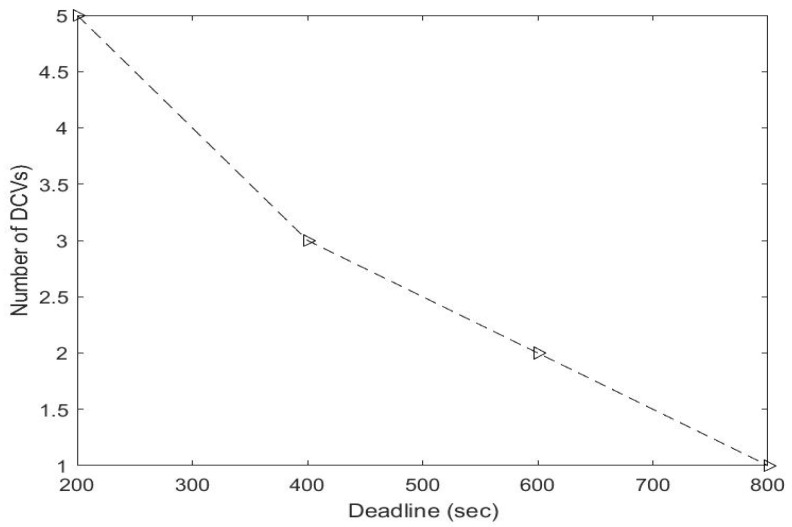
Number of DCVs at specific deadlines.

We set the deadline time to accomplish data collection to 400 s. Figure 12 shows the result of the maximum time usage with different numbers of MCVs. The figure shows that when n>3 (with more than 3 DCVs), the maximum time usage is less than 400 s. This implies that all DCVs would be able to complete their tasks before the deadline when the number of DCVs is greater than two. Thus, the optimal number of DCVs in this case is three. Figure 13 shows the result of the optimal number of DCVs at specific deadlines. 

### 5.3. Optimizing Path Planning Strategy of DCVs

The trajectory planning of the DCV is designed to meet certain deadline time requirements that guarantee the efficiency and usefulness of the collected data and minimize the energy-hole problem in the network. An energy-hole problem results when the energy of a sensor node has fallen below the threshold, making continuous data sensing operations difficult. The energy-hole problem can be minimized by achieving timeliness in data collection and transmission. Each DCV can be positioned at a specified location on an optimal path, which is calculated using Equation (12). We employ the nearest-neighbor (NN) heuristic algorithm presented in [63] to obtain the optimal trajectory. To this end, a DCV begins from the initial location and traverses along a specified path, only stopping at designated points for data collection, and returns to its initial location after each tour. This process is repeated until all the DCVs have completed their tasks. The time taken for a DCV to accomplish its task depends on the number of regional optimal points. To optimize time and energy consumption, each DCV is assigned an average number of regional optimal points closest to them. 

The proposed approach deals with a multi-routed problem to determine optimal *Q* routes ($R_{1},\;R_{2},\;\ldots ,\;R_{Q}$) to collaboratively visit all z optimal points, such that the longest time taken to traverse the Q routes is minimized. We note that data gathering using multiple mobile elements is the same as the multiple traveling salesman problem (TSP) [64], and by adapting the approaches presented in [11,65], it can be shown to be an *NP*-hard problem. Thus, heuristic algorithms can be employed to achieve a near-optimal solution. To achieve the deadline time constraint and optimize energy consumption, we determine an optimal route for each DCV and assign an average number of optimal points to each DCV. Equation (13) helps to calculate the average number of optimal points assigned to each MCV.

(13) ANOP(MCV)=sV 

where $A_{NOP}$ denotes the average number of optimal points assigned to each DCV, *s* represents the number of optimal points, and *V* denotes the number of DCVs. In situations in which the number of optimal points (*s*) is not evenly divisible by *V*, Equation (14) can be employed.

(14) sV=Q, …, R(for V DCVs, R=Q+1 and else Q)

Equation (14) implies that *R* DCVs must traverse a *Q* + 1 number of optimal points, while the others must traverse *Q* number of optimal points. While *Q* represents the quotient of s/V, *R* represents the remainder. For instance, if three DCVs are deployed to eight optimal points according to Equation (14), this means that *s* = 8, *V* = 3, *Q* = 2, and *R* = 2. Therefore, the allocation of eight optimal points to three DCVs will take the form of [3; 3; 2]. The number of optimal points (OPs) allocated to each DCV can be computed using a distance matrix whereby the distances between the OPs and all DCVs is represented by the following entries:

(15) $$d_{matrix}\left(DCVs,\;OP_{s}\right)=\left[\begin{array}{c} d_{11}\;d_{12}\;d_{13}\;\ldots \;d_{1s}\\ d_{21}\;d_{22}\;d_{23}\;\ldots \;d_{2s}\\ d_{31}\;d_{32}\;d_{33}\;\ldots \;d_{3s}\\ \ldots \;\ldots \;\ldots \;\ldots \;\\ d_{V1}\;d_{V2}\;d_{V3}\;\ldots \;d_{Vs} \end{array}\right]\;$$

where the number of DCVs is the row index, while that of the OPs is the column index. The number of OPs allocated to a DCV is determined by the minimal value of the distance matrix in the respective column. For instance, an $OP_{2}$ is assigned to $DCV_{2}$ if the distance between it and $DCV_{2}$ is less than that from other DCVs. The same goes for all other OPs and the relevant DCVs. That is, if $d\left(DCV_{2},OP_{2}\right)<d\left(DCV_{1},\;OP_{2}\right)$, then $OP_{2}\to DCV_{2}$. In general, an OP is randomly assigned to any one of the multiple DCVs if the distance between them is minimum. Each $DCV_{i}$ begins its tour from the starting location, traverses all OPs in $OP_{i}$, and finally heads back to the starting point. The total path length traversed by a $DCV_{i}$ can be computed using Equation (16):

(16) $$L_{path\left(\vartheta i\right)}=d\left(DCV_{i},OP_{i}\right)+\overset{NOP_{i}-1}{\underset{k=1}{\sum }}\left(OP_{ik},\;OP_{ik+1}\right)+d\left(OP_{iNop},\;DCV_{i}\right)\;$$

where 1≤i, k≤V and i≠k, $nOP_{i}$ denotes the number of OPs on the ith path.

## 6. Simulation Experiments and Performance Analysis

The performance of the proposed approach was simulated in the MATLAB environment using RMASE, an application platform running under PROWLER. The simulator, as an event-driven WSN simulator, utilizes codes that are compatible with those of MATLAB. RMASE has well-defined metrics, the values of which are generated as the simulation is being run. The simulations are conducted considering various numbers of sensors, cluster heads, and optimal points located in a network with a size of 1000 × 1000 square meters. The nodes are deployed randomly, each with an initial energy of 2J. The DCVs have no energy constraints and can move at a speed of 10m/s. Our experiment presents an improved energy-efficient ant-based routing (IEEABR), which is compared with three other swarm-intelligence-based routing protocols namely, Energy-Efficient Load Balancing Ant-based Routing Algorithm (EBAR) [66], Bee-Sensor-C [67], and Flooded Forward Ant-based Routing (FF) [68]. The performance of the proposed approach was tested with the aforementioned algorithms using some evaluation metrics. We note that modeling IoT-based systems as swarm intelligence comes with some additional benefits that allow such systems to share in the benefits inherent in swarm intelligence systems, such as robustness, flexibility, and scalability [54]. These major features have the potentials to help the IoT-based system to overcome its main architectural challenges, such as scalability, robustness, flexibility, and interoperability [69]. A summary of the simulation parameters is presented in Table 4.

### 6.1. Simulation Experimental Data Analysis

**Figure 14:**
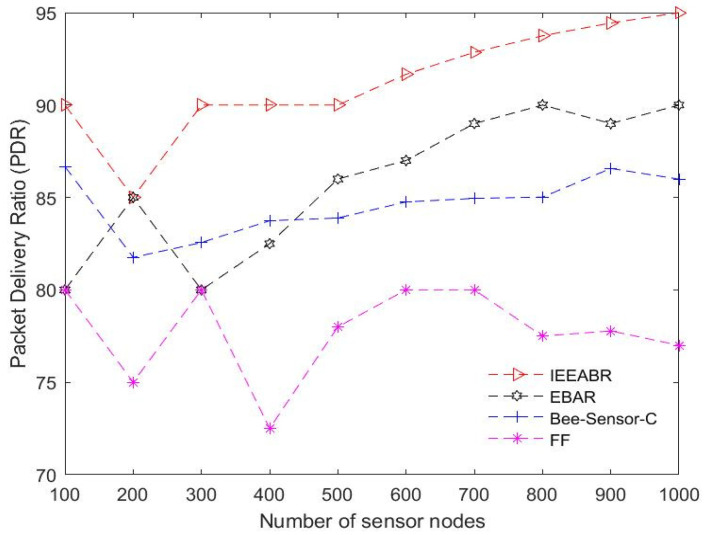
Comparison based on the packet delivery ratio.

**Figure 15:**
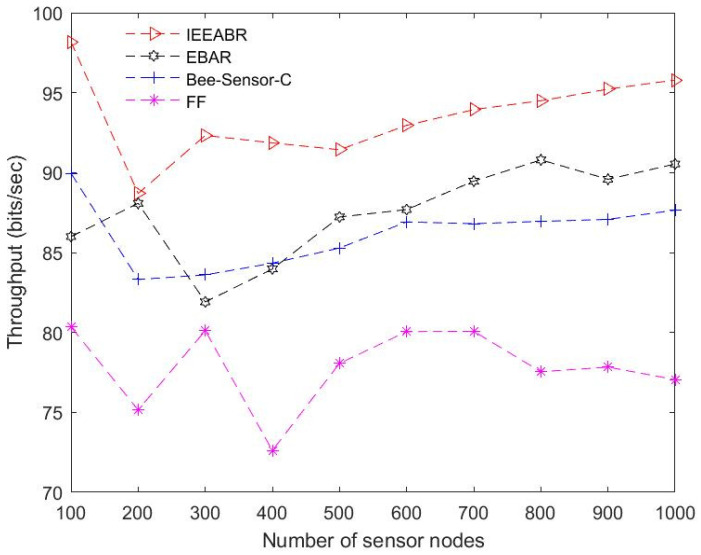
Comparison in terms of throughput.

**Figure 16:**
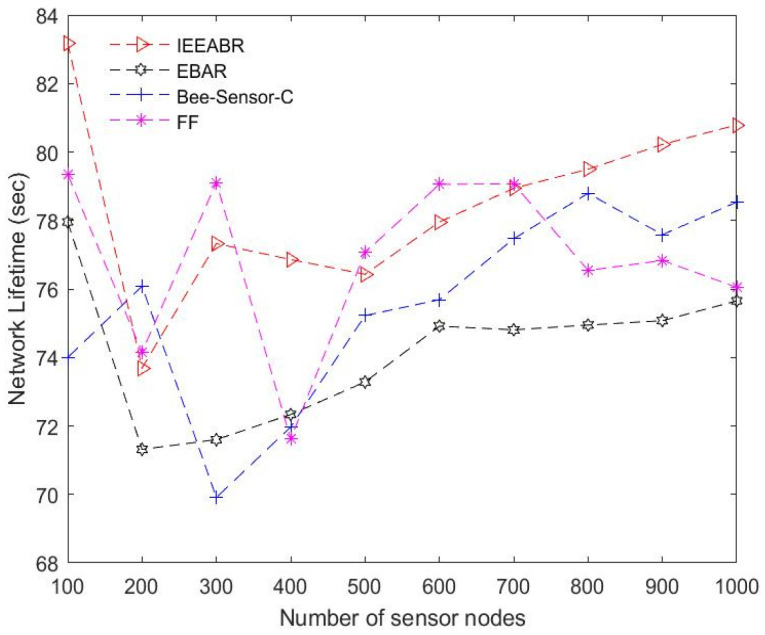
Comparison in terms of network lifetime.

**Figure 17:**
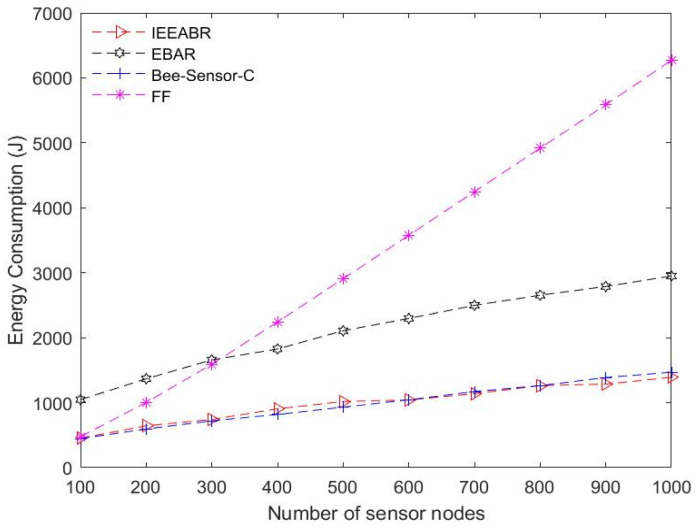
Comparison based average energy consumption.

**Figure 18:**
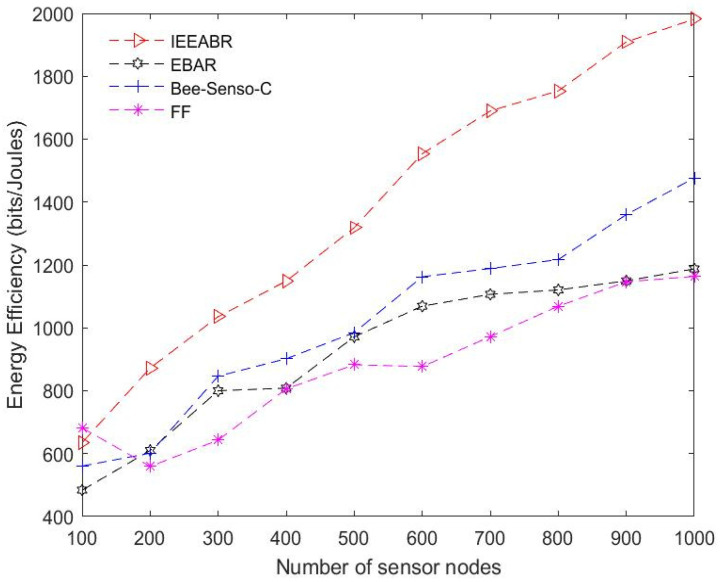
Comparison in terms of energy efficiency.

**Figure 19:**
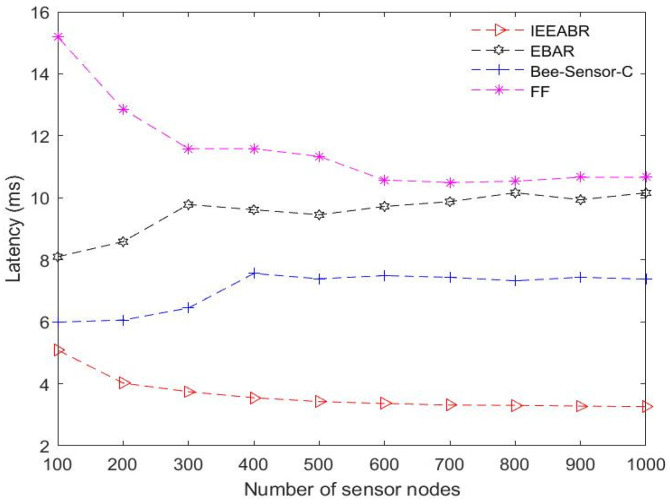
Comparison based on latency.

Table 5 is a comparison table that shows a summary of the numerical analyses conducted for the results provided in Figure 14, Figure 15, Figure 16, Figure 17, Figure 18 and Figure 19. The table result is an average of 10 simulation runs performed for each of the routing protocols using the various evaluation metrics. In general, the results show that the IEEABR outperformed the other protocols in terms of the considered evaluation metrics, showing the efficacy of swarm intelligence (SI)-based approaches.

### 6.2. Results Analyses

Figure 14 shows the performance of the different SI-based routing protocols in terms of the packet delivery ratio (PDR). PDR is referred to as the ratio of the total number of packets transmitted and received at the sink node or base station (BS). The total number of transmitted packets at the BS may be lower than or equal to those generated at the sensor nodes in the network. The results show that IEEABR outperformed the other SI-based protocols. The results show that initially, at a very minimal node density, IEEABR performed optimally by delivering approximately 90% of all the generated packets in the network at the BS via the DCVs, followed by Bee-Sensor-C, which achieved approximately 87% PDR, while EBAR and FF both achieved 80% PDR. As the node density gradually increased, IEEABR maintained optimal performance compared to the other protocols and achieved 95% PDR at a maximum node density of 1000. However, the performance of Bee-Sensor declined as the node density gradually increased and was outperformed by EBAR, which achieved 90% PDR at a maximum node density of 1000, followed by Bee-Sensor, with 86% PDR at a node density of 1000. The high performance of IEEABR is significant for critical large-scale sensor network applications running in smart cities, which require high data delivery performance. This implies that waste management operations employing IEEABR strategies can achieve high delivery performance to meet the requirements of the smart city ecosystem.

Figure 15 shows the performance of the SI-based protocols in terms of throughput. Throughput refers to the overall number of packets that are successfully delivered at the sink node or BS within one second. In general, IEEABR outperformed the other routing protocols, followed by EBAR, Bee-Sensor-C, and FF. An important observation in Figure 14 is the performance decline in throughput values with increasing node density. Ordinarily, throughput is improved by increasing the number of sensor nodes in the network. However, increasing the node density in the network should be synchronous with the number of DCVs sent into the network. However, due to the issue of network complexity and cost, increasing the number of DCVs as the node density increases is counterproductive, requiring an optimal number of DCVs to maintain network stability. Hence, the decline in throughput values exhibited by IEEABR, Bee-Sensor-C, and FF with increasing node density is normal.

Figure 16 shows the network operational lifetime prediction of the routing protocols. The network lifetime is the extended lifetime of the network for various rounds in a given time. As can be observed, IEEABR outperformed the other protocols, with the longest lifetime. All the routing protocols achieved good network lifetime, with IEEABR achieving the longest network lifetime, followed by Bee-Sensor, EBAR, and FF. This evaluation is very significant, as it shows the efficacy of SI-based techniques in saving energy and prolonging the network lifetime. This makes such techniques suitable for SC applications such as waste management and target-tracking applications. Applications employing SI-based techniques for data collection are able to minimize energy consumption at the sensor nodes since data collection within short ranges is enabled, and protocol overheads are transferred to the DCVs, thereby saving energy at the sensor nodes, optimizing their energy consumption and increasing the network lifetime.

Figure 17 and Figure 18 show the performance in terms of the average energy consumption and energy efficiency, respectively. The energy consumption of the sensor nodes refers to the total energy expended by all sensor nodes in the network in transmitting their data to the DCVs. In this case, IEEABR outperformed the other protocols for these metrics by achieving a low energy consumption, even at a maximum node density, and achieving the highest energy efficiency, followed by Bee-Sensor-C, EBAR, and FF. This suggests that applications employing IEEABR techniques minimize their energy consumption values and optimize energy efficiency. Optimizing energy consumption for big data collection in LS-WSNs is one of the critical targets of this study, which proposes an SI IoV-based technique for data collection for SC waste management applications. It can be seen that this objective can be clearly achieved by employing SI-based techniques exploiting IEEABR strategies.

Figure 19 shows the performance in terms of latency. The latency in this context refers to the time required for a DCV to traverse the OPs/DCPs for data gathering and transferal of the collected data to the BS. However, the time spent by the CHs on data gathering within their clusters is negligible. This is because data transmission speed within clusters is faster than that between CHs and DCVs, so the data gathering latency is mostly affected by the trajectory length. Hence, in determining the latency, there is a need to consider the total number of location points or DCPs. As shown in Figure 19, IEEABR outperformed the other protocols on this metric by achieving the lowest latency compared to the other models, even at a maximal node density, followed by Bee-Sensor-C, EBAR, and FF. The reason for the high latency of FF with a potential high energy consumption may be its redundant message retransmissions of data and frequent message collisions in a dense network, as well as flooding of the network, resulting in network congestion. However, waste management scenarios may not involve latency as a major issue since the vehicles are exploited opportunistically for data collection within some degree of delay tolerance.

In general, SI-based protocols are preferrable for real-time applications such as environmental monitoring applications, including detection of waste disposal violations [70], target tracking scenarios (event-driven), and surveillance operations [2,71,72,73]. This is because such protocols have potential for a high data delivery rate per unit of energy consumption compared to their classical counterparts and therefore generate fewer overheads and optimize energy consumption. The performance of the SI-based routing protocols shows that the proposed system exploiting an SI IoV-based method for data collection for SC waste management is efficient, especially for LS-WSN scenarios.

## 7. Conclusions

In this paper, we presented swarm intelligence IoV-based approaches for opportunistic data collection and traffic engineering in SC waste management. The proposed opportunistic approach for data collection using SI-based methods employing vehicles with predetermined routes is aimed at minimizing latency times and optimizing energy consumption. In this study, we proposed analytically based methods to investigate critical tradeoffs in optimizing energy consumption for big data collection and transmission in an LS-WSN, such as finding the optimal number of DCVs required in the network to maintain network stability and determining the optimal number of DCPs for the DCVs. These critical tradeoffs have not been addressed in previous studies exploring waste management techniques for SCs. Unlike many existing techniques, we envisioned methods employing SI-based approaches for big data collection for SC waste management operations. Towards this objective, we compared some SI-based routing protocols using evaluation metrics. The results of this evaluation show the efficacy of SI-based routing protocols in terms of scalability, flexibility, and robustness, confirming the feasibility of the proposed approach to be exploited to optimize energy consumption for big data collection in SC waste management scenarios. Future research directions will be channeled towards exploiting SI-based techniques to determine the mobility patterns of vehicles.

## Figures and Tables

**Table 1:** Summary of previous works on IoT-based strategies for waste management in an SC.

IoT-Based Technique/Approach	Applications	Feature/Focus of Work	Ref.
A stochastic optimization scheme to improve waste recovery and collection processes	Health, entertainment, education, and urban living	Reduction of operational costs, energy dissipation, transportation and pollution emissions	[23]
A novel algorithm providing effective and scalable solutions for dynamic waste collection	Health, education, urban living, gas stations, and factories	Optimizing routing functionalities for several trucks	[24]
Application of data envelopment analysis (DEA) to quantify and benchmark waste generation intensity	Machine, energy, internet networks, logistics, mobile devices, and monitoring	Real-time benchmarking analysis of waste monitoring to realize waste reduction potentials on the floor of a manufacturing shop	[25]
IoT-based technologies (Android application, cloud, GSM/GPRS, sensors, etc.)	Workplace, GSM, logistics, sensors, surveillance, GPS, RFID, and urban living	Providing efficient waste collection solutions and economic feasibility and obtaining real-time information about the status of dustbins	[26,27,28,29,30]
A cloud-based advanced decision support system (DSS) for dynamic scheduling and routing models	Automation, RFID, monitoring, sensors, and surveillance cameras	Efficient waste collection and dynamic route optimization in SCs	[31,32,33,34]
Simulation-based approach using load cell sensors	Load cell sensors, GPS, GSM, and Web applications	Determinining the maximum waste load capacity inside waste bins	[35]
A robust counter model of an open capacitated vehicle routing problem	Workplace	Determining the optimal routes for garbage transportation	[36]
IoT-based approach for real-time data collection for waste management in an SC	Urban living, GPRS, RFID tags, and volumetric ultrasonic sensor	Optimizing waste collection routes and methods to minimize envorinmental hazards	[37]
IoT-based automated machine learning approach	Raspberry Pi, GPRS, ultrasonic sensors, and urban environments	Real-time waste monitoring and collection and optimization of time costs and operational efficiency	[38,39,40,41]
Application of big data analysis to vehicle routing using GPS data	GPS and big data	Optimizing vehicle routing and waste collection	[42]
A smart dual dustbin model using ultrasonic & IR sensors, GSM, Servo Uno, Arduino	Ultrasonic sensors, IR obstacle sensors, GSM, Servo, and Arduino	Efficient waste mgt. with advanced dynamic routing and optimized routes	[43]
A Smart (IoT-Based) dustbin model using IR sensors LCD	IR sensors, GSM, and monitoring systems	Garbage monitoring and control	[44]
IoT-based smart garbage monitoring and clearance alert system using RGB LED, ultrasonic sensors, GSM, GPS, and Wi-Fi	LED lights, sensors, GSM, Arduino, GPS, and Wi-Fi	Garbage-level monitoring for disposal, clearance, routing optimization; detecting the max. capacity level of garbage	[45,46]
Application of a lab-scale landfill model	Sensors and LM35 temp. sensors	Monitoring and processing of food waste	[47]

**Table 2:** Comparison of the proposed approach with other SI-based techniques.

Ref.	Approach		Application in SC Waste Management	IoV-Based Approach		SI-Based Approach	Energy Efficiency
	Stochastic Optimization	Metaheuristic		Yes	No		
[11]	*	*			*	*	*
[48]		*			*	*	
[50]		*			*	*	
[52]		*			*	*	*
[54]		*			*	*	
[55]	*	*			*	*	
[56]	*	*			*	*	*
[57]	*	*			*	*	*
[58]		*			*	*	*
Proposed approach	*	*	*	*		*	*

**Table 3:** Notations with their descriptions.

Symbol	Description
*N*	The number of sensor nodes
*g*	Number of groups in the network
*V*	Optimal number of data collector vehicles (DCVs)
*h*	The total number of cluster heads (CHs)
Ć	A set of all cluster heads (CHs)
$r_{k}$	Transmission range of sensor nodes
*r*	Total number of overlapped regions
*s*	Total number of optimal points
$T_{m}$	Total movement time of the data collector vehicle
$\Omega_{OP}$	A set of all optimal location points
$OP_{i}$	*i*th subset of $\Omega_{OP}$
v	Moving speed of a DCV
$nOP_{i}$	Number of optimal points in $OP_{i}$
$OP_{inOP_{i}}$	Number of optimal points assigned to $DCV_{i}$
$OP_{j}$	An optimal location point, 1≤j≤s
Ŕ	A set of all regions
*L*(*ϑ*)	The length of traveling path *ϑ*
$L_{path\left(\vartheta i\right)}$	Total path length traveled by $DCV_{i}$
$T_{d}$	Total time spent by a DCV in one trip
$T_{so}$	Stopping time at an optimal point
$T_{opt}$	Optimal time
$T_{c}$	Deadline time constraint for data collection

**Table 4:** Simulation parameters.

Parameter	Value
Simulator	RMASE
Network size	1000 × 1000 m^2^
Node deployment	Random
Number of sensor nodes	100–1000
Transmission range ($r_{k})$	35 m
Data packet size	512 bytes
Time deadline ($T_{c})$	400 s
Data transmission rate	250 kbps
Collection rate of DCV	40k bps
DCV speed	10 m/s
Initial energy ($E_{so})$	2.0 J
MAC	802.11
Radio propagation model	Two-Ray Ground
Channel model	Wireless channel
Antenna	Omni-Antenna
$E_{elec}$	50 nJ/bit
$E_{fs}$	10 pJ/bit/m^2^
$E_{mp}$	0.0013 pJ/bit/m^4^
Simulation time	1500 s
Initial position of DCV	(0,0)

**Table 5:** Summary of simulation results of routing protocols using different metrics.

Routing Algorithms				
	IEEABR	EBAR	Bee-Sensor-C	FF
Packet delivery ratio (%)	91.2714	85.85	84.5911	77.778
Throughput (bits/s)	93.4888	87.5245	86.188	77.8831
Network lifetime (sec)	78.4888	74.188	75.5245	76.8831
Energy consumption (J)	1389.6	2950.0	1468.2	6271.2
Energy efficiency (bits/J)	1981.87	1187.5	1476.67	1163.55
Latency (ms)	3.2637	10.161	7.375	10.663

## Data Availability

Not applicable.
